# Frequent discordance between clinical and musculoskeletal ultrasound examinations of foot disease in juvenile idiopathic arthritis observed in the multidisciplinary setting

**DOI:** 10.1186/1757-1146-4-S1-O19

**Published:** 2011-05-20

**Authors:** Gordon J Hendry, Martijn PM Steultjens, Janet Gardner-Medwin, Jim Woodburn, Debbie E Turner

**Affiliations:** 1 School of Biomedical and Health Sciences, University of Western Sydney, Sydney, Locked Bag 1797, Australia; 2 Glasgow Caledonian University, Glasgow, G4 0BA, UK; 3 University of Glasgow, Glasgow, G12 8QQ, UK

## Background

Ultrasound has shown promise for detection of sub-clinical disease in JIA. This may be particularly beneficial for the foot and ankle joints, which are difficult to examine in children. Early detection of sub-clinical foot disease permits earlier intervention which may improve outcome. The aim of this study was to evaluate agreement between clinical and ultrasound examinations of foot disease in JIA.

## Methods

Thirty patients with JIA underwent clinical and US examination of 24 foot joints, 10 tendons and 6 peri-articular soft tissues. Each site was examined independently by a rheumatologist and a podiatrist for synovitis, and tenderness/swelling. At the same sites the sonographer examined independently for effusion, synovial hypertrophy, power Doppler signal (PS), tenosynovitis, or abnormal tendon thickening. Agreement was estimated using Cohen’s unweighted kappa (κ) (>0.4 = moderate agreement) with associated 95% confidence intervals.

## Results

720 joints, 300 tendons and 180 soft tissue sites were assessed. Clinically detected synovitis, tenderness and swelling were recorded in 42 (5.8%), 78 (10.8%) and 73 (10.1%) joints respectively. US-detected effusions, synovial hypertrophy and PS were recorded in 88 (12.2%), 47 (6.5%) and 12 (1.7%) joints. Tenderness and swelling were recorded in 29 (9.7%) and 16 (5.3%) tendons and 28 (15.6%) and 9 (5%) soft tissues. US-detected tenosynovitis and PS were detected in 7 (2.3%) and 6 (2%) tendons. Abnormal thickening of the plantar fascia origin and Achilles tendon insertion were detected at a frequency of 4/60 (6.7%) and 1/60 (1.7%), and 3/60 (5%) effusions were recorded at the retro-calcaneal bursa. Subclinical foot disease was discovered in 52 (7.2%) joints, 5 (1.6%) tendons and 4 (2.2%) soft tissue sites. Agreement was consistently less than moderate (κ<0.4) for each clinical and US interaction. There was moderate agreement between the rheumatologist and podiatrist for active synovitis versus joint swelling (κ=0.52).

## Conclusions

There is frequent discordance between clinical and US assessments of foot disease in JIA. Subclinical foot disease appears common; however clinical examination also detected features of active disease in structures that were recorded as normal on US. These findings suggest US may be a useful tool to aid clinical examination of the foot in JIA patients.

